# The RFTS Domain of Raf2 Is Required for Cul4 Interaction and Heterochromatin Integrity in Fission Yeast

**DOI:** 10.1371/journal.pone.0104161

**Published:** 2014-08-04

**Authors:** Sharon A. White, Alessia Buscaino, Luis Sanchez-Pulido, Chris P. Ponting, Matthew W. Nowicki, Robin C. Allshire

**Affiliations:** 1 Wellcome Trust Centre for Cell Biology and Institute of Cell Biology, School of Biological Sciences, The University of Edinburgh, Edinburgh, Scotland, United Kingdom; 2 School of Biosciences, Kent Fungal Group, University of Kent, Canterbury, Kent, United Kingdom; 3 MRC Functional Genomics Unit, Department of Physiology, Anatomy and Genetics, University of Oxford, Oxford, United Kingdom; St Jude Children's Research Hospital, United States of America

## Abstract

Centromeric heterochromatin assembly in fission yeast is critical for faithful chromosome segregation at mitosis. Its assembly requires a concerted pathway of events whereby the RNA interference (RNAi) pathway guides H3K9 methylation to target sequences. H3K9 methylation, a hallmark of heterochromatin structure, is mediated by the single histone methyltransferase Clr4 (equivalent to metazoan Suv3-9), a component of the CLRC complex. Loss of or defects in CLRC components disrupts heterochromatin formation due to loss of H3K9 methylation, thus an intact, fully functional CLRC complex is required for heterochromatin integrity. Despite its importance, little is known about the contribution of the CLRC component Raf2 to H3K9 methylation and heterochromatin assembly. We demonstrate that Raf2 is concentrated at centromeres and contrary to other analyses, we find that loss of Raf2 does not affect CENP-A^Cnp1^ localisation or recruitment to centromeres. Our sequence alignments show that Raf2 contains a *R*eplication *F*oci *T*argeting *S*equence (RFTS) domain homologous to the RFTS domain of the human DNA methyltransferase DNMT1. We show that the Raf2 RFTS domain is required for centromeric heterochromatin formation as its mutation disrupts H3K9 methylation but not the processing of centromeric transcripts into small interfering RNAs (siRNAs) by the RNAi pathway. Analysis of biochemical interactions demonstrates that the RFTS domain mediates an interaction between Raf2 and the CLRC component Cul4. We conclude that the RFTS domain of Raf2 is a protein interaction module that plays an important role in heterochromatin formation at centromeres.

## Introduction

RNAi is a widely conserved process in eukaryotes characterised by small RNAs bound by Argonaute effector proteins which act as guides to target homologous sequences for repression [1], [2], [3]. RNAi can act post-transcriptionally to regulate gene expression either by translational inhibition or transcript cleavage [4]. In addition, RNAi can also mediate DNA and chromatin modifications which cause transcriptional silencing and heterochromatin formation [5]. RNAi-directed heterochromatin formation is critical for centromere function in the fission yeast, *Schizosaccharomyces pombe* [6]. This process is well characterised in *S. pombe* due in to its genetic tractability and the fact that it encodes only single non-essential genes involved in this pathway [6].

In fission yeast, the main domains of heterochromatin are found at centromeres, telomeres and the silent mating-type locus [6], [7]. Despite the fact that marker genes inserted within centromeric repeats are transcriptionally silenced, it is known that the repeats themselves are bi-directionally transcribed by RNA polymerase II (RNAPII) during S phase [8], [9], [10], [11], [12]. These non-coding centromere transcripts generate double-stranded RNA (dsRNA) which is processed by the ribonuclease enzyme Dicer into 22–25 bp small interfering RNAs (siRNAs). Centromeric siRNAs act to guide the Argonaute/Ago1 effector protein, a component of the RNA-Induced Transcriptional Silencing (RITS) complex, to homologous sequences. The RITS complex binds chromatin via the chromodomain protein Chp1 and recruits the CLRC complex to the centromeric repeats via the linker protein Stc1 [13], [14], [15]. Clr4, a component of the CLRC, is the only histone methyltransferase which methylates H3 on lysine 9 in fission yeast. H3K9 methylation (H3K9me) creates a binding site for the chromodomain proteins Swi6, Chp1 and Chp2, which are required for the spreading of heterochromatin and the binding of RITS to chromatin [16], [17], [18]. Swi6 and Chp2 are orthologs of HP1 (heterochromatin protein 1) which binds H3K9 methylated chromatin in metazoa.

In addition to Clr4, the CLRC complex consists of the cullin scaffold protein Cul4, the β-propeller protein Rik1, the RING box protein Rbx1, the WD-40 protein Raf1/Dos1 and Raf2/Dos2 [19], [20], [21], [22]. We have previously shown that members of the CLRC complex (Cul4, Rik1, and Raf1) are predicted to adopt a structure similar to the conserved Cul4-DDB1-DDB2 E3 ubiquitin ligase and recent structural analysis of Raf1 has confirmed this prediction [23], [24]. In addition, the CLRC complex has been shown to possess E3 ligase activity *in vitro* [19].

Although CLRC has been the subject of extensive study, the role of the Raf2 subunit within this complex and its contribution to heterochromatin formation remains elusive.

In addition to its association with CLRC, Raf2 has been proposed to regulate transcription within heterochromatin during S phase via its interaction with Cdc20 (DNA polymerase-ε) and the transcription factor Mms19 at replication forks [25]. Moreover, Raf2 has recently been implicated in the localisation of the CENP-A^Cnp1^ histone H3 variant to centromeres [26]. Raf2 contains a C2H2 type zinc finger and an N-terminal region which exhibits similarity to the Replication Foci Targeting Sequence (RFTS) domain found in the DNA methyltransferase DNMT1. The RFTS domain is conserved across fungi, plants and animals but only the RFTS domain of mammalian DNMT1 has been characterised [27].

DNMT1 is the major enzyme responsible for the maintenance of the typically repressive DNA modification in plant and vertebrate cytosine methylation of CpG dinucleotides [28]. The RFTS domain of DNMT1 has been implicated in its catalytic function, protein interactions and subcellular localisation [29], [30], [31], [32]. The UHRF1 protein interacts with the RFTS domain of DNMT1. UHRF1 is a multi-domain protein (UHRF: ubiquitin-like, containing PHD and RING finger domains 1) with E3 ligase activity, which has been shown to be required for the degradation of DNMT1 and can bind to histone H3 methylated on lysine 9 [31], [33], [34]. Consequently, UHRF1 mediates cross talk between DNA methylation and post-translational modification of histones, specifically H3K9 methylation [34]. Thus a clear link between the RFTS domain of DNMT1, an E3 ligase and chromatin modification has been established; it is likely that RFTS domains mediate similar interactions in other eukaryotes.

We set out to characterise the RFTS domain of Raf2 and its role in centromeric heterochromatin formation. We show that the RFTS domain of Raf2 can be modelled on that of DNMT1 and that specific residues within this domain are crucial for heterochromatin integrity. We demonstrate that alteration of particular residues within the RFTS domain disrupts a direct interaction between the Raf2 and the Cul4 subunit of CLRC. Furthermore, although heterochromatin is disrupted, the generation of siRNA remains unperturbed, suggesting that Raf2 has separable roles in chromatin modification and siRNA production. Thus we have identified the RFTS domain of Raf2 as a protein interaction module crucial for heterochromatin integrity and centromere function.

## Materials and Methods

### Strain and plasmid construction

Standard procedures were used for bacterial, fission yeast and budding yeast growth and genetic manipulations [35]. *S. pombe* strains used in this study are described in Table S1. Primer sequences are listed in Table S2.

Deletion and epitope tagging (3xFLAG) of Raf2 was achieved by homologous recombination with PCR fragments comprising resistance cassettes flanked by sequence homologous to insertion sites [36]. *Raf2-I98A* and *Raf2-E104A* mutations were generated by mutagenizing pDONR201-Raf2 using the QuikChange XL Site-Directed Mutagenesis Kit (Stratagene). Raf2 mutant PCR products were generated using primers with 80 bp homology to the each side of the site of recombination and transformed into the FY17087 strain bearing the RFTS domain replaced with a *ura4^+^* marker gene. Correct integrants were selected on FOA media and confirmed by PCR and sequencing of the *raf2^+^* gene.

The *Raf2-S100F* allele was isolated in a random UV mutagenesis genetic screen. FY 1181 cells were spread on YES plates lacking adenine, irradiated with 15000 µJ (around 50% killing) and incubated at 36°C for 5–7 days. Fast-growing colonies were picked and tested for thermosensitivity of silencing at *otr1R(SphI):ade6^+^* and for supersensitivity to TBZ. Mutants were backcrossed at least three times. *Raf2-S100F* mutation was identified by complementation and sequencing of the *raf2^+^* gene.

### Structural modeling and alignments

Sequence and secondary structure alignments were produced using Jalview version 6.1 using Muscle, a multiple protein sequence alignment method [37]. The model of Raf2 RFTS domain was produced via alignment to the RFTS domain of murine DNMT1 (PDB code 3AV4), using Phyre2 in intensive mode, 167 residues (89%) modeled at >90% accuracy [38]. Alignments are shown to murine DNMT1 (3AV4, [39]) and human DNMT1 (3EPZ, [27]).

### Cytology

Immunostaining was performed as described previously [40]. Cells were fixed with 3.7% PFA/10 min, plus 0.05% glutaraldehyde for tubulin staining. Antibodies used were TAT1 anti-tubulin 1∶15 (gift from K. Gull), anti-Cnp1 1∶2000 and anti-GFP 1∶200 (A11222, *Life Technologies*). Alexa Fluor 594- and 488-coupled secondary antibodies were used at 1∶1000 (*Life Technologies*). Microscopy was performed using a Zeiss Imaging 2 microscope using a 100× 1.3 NA Plan-Apochromat objective. Image acquisition was controlled using Metamorph software (*Universal Imaging Corporation*). Identical exposures were used for different strains in the same experiment. For co-staining experiments, cells were visually scored for a single interphase Cnp1 cluster at centromeres and with/without Raf2 or Swi6 co-staining were counted.

### Chromatin immunoprecipitation

Chromatin immunoprecipitation (ChIP) was performed as described with the following modifications [41]. Cells were fixed in 1% PFA/15 min for H3K9me2 and Cnp1 ChIP. One microliter of monoclonal H3K9me2 antibody (m5.1.1) and 10 microliters of α-Cnp1 antiserum was used per ChIP. Real-time PCR (qPCR) was performed using the LightCycler 480 SYBR Green I Master (*Roche*) on a LightCycler 480 Instrument (*Roche*). qPCR analysis primers are shown in Table S2. Relative enrichments were calculated as the ratio of product of interest to control product (*act1*+) in IP over input. Histograms represent data from three biological replicates analyzed in parallel.

### RNA analysis

Northern analysis of centromeric siRNAs and qRT-PCR analysis of centromeric transcripts were performed as described previously [42]. siRNA probes and primers for qRT-PCR are listed in Table S2.

### Yeast-2-hybrid

Yeast two-hybrid vectors were generated by PCR amplification of the Raf2 ORF with primers bearing SalI and BamHI sites. Purified Raf2 PCR products were digested and cloned into SalI/BamHI digested pGBKT7 (Clontech ‘Matchmaker’ system). Plasmids pGAD-*Raf2-I98A*, pGAD-*Raf2-E104A* and pGAD-*Raf2-S100F* were generated with QuikChange XL Site-Directed Mutagenesis Kit (Stratagene). The yeast-2-hybrid assay was performed as described previously [23].

## Results

### Raf2 localises to centromeric heterochromatin repeats but does not affect CENP-A localisation to heterochromatin

**Figure 1:**
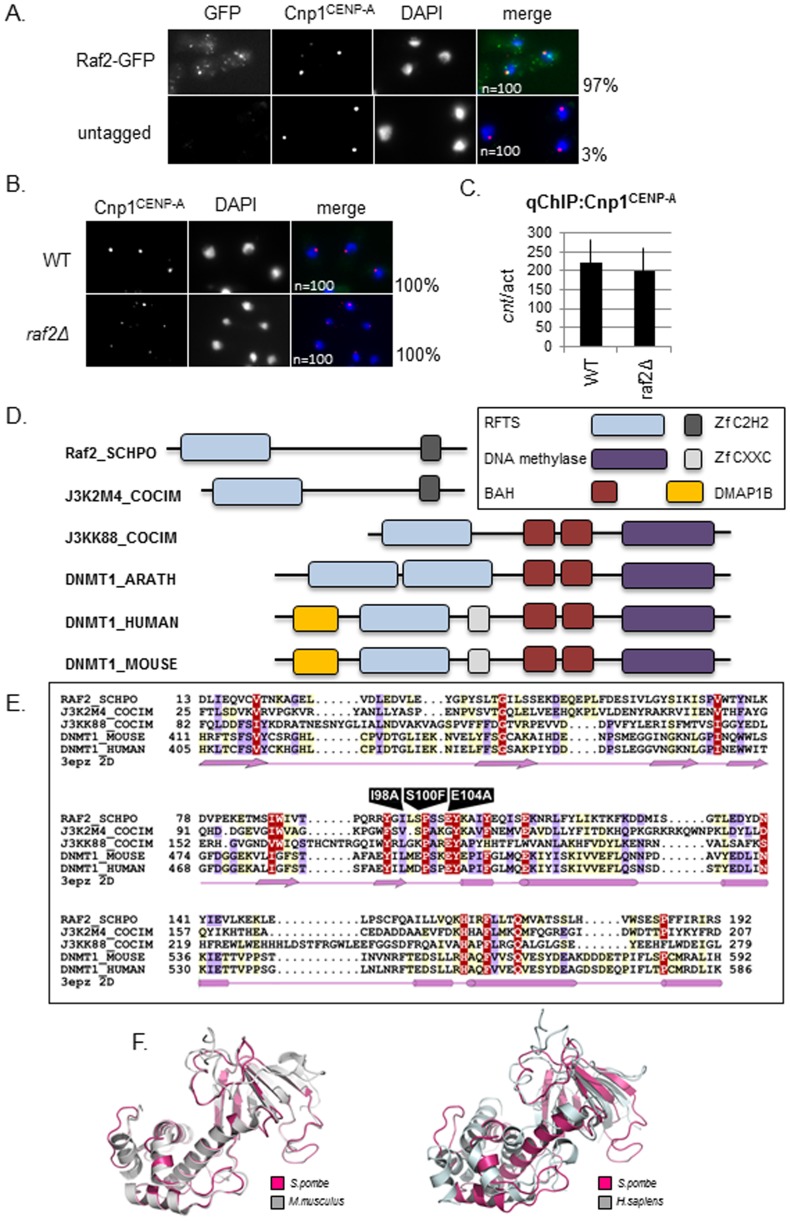
Raf2 is an RFTS domain protein and localises to the nucleus. **A.** Analysis of Raf2 localisation in wildtype cells by immunofluorescence. Raf2 localises predominantly to the nucleus and colocalises with Cnp1 at centromeres (numbers indicate % cells displaying colocalisation n = 100). Representative images show staining of fixed cells for Raf2-GFP (green), Cnp1 (red) and DNA (DAPI-blue). **B.** Analysis of Cnp1 localisation in *raf2Δ* cells. Cnp1 remains localised in cells lacking Raf2 (numbers indicate % cells displaying localisation n = 100). Representative images show staining of fixed cells for Cnp1(red) and DNA (DAPI-blue). **C.** ChIP analysis of Cnp1 levels in *raf2Δ* cells. Cnp1 remains associated with the central core domain in cells lacking Raf2. Error bars indicate standard error of the mean (SEM). **D.** Schematic diagram showing the domain architectures of Raf2 and DNMT1 families. Sequences are named according to their UniProt names. Full species names are: RAF2_SCHPO, *Schizosaccharomyces pombe*; J3K2M4_COCIM, *Coccidioides immitis*; J3KK88_COCIM, *Coccidioides immitis*; DNMT1_ARATH, *Arabidopsis thaliana*; DNMT1_HUMAN, *Homo sapiens*, DNMT1_MOUSE, *Mus musculus*. E. Multiple sequence alignment of RFTS domain. The human DNMT1 RFTS domain secondary structure (pdb: 3epz) is shown below. Arrows and cylinders depict sheets and helices respectively. Residues which are subject to mutation are labeled. The amino acid colouring scheme indicates average BLOSUM62 scores (which are correlated with amino acid conservation) for each alignment column: red (greater than 2), violet (between 2 and 1) and light yellow (between 1 and 0.2). Sequences are named according to their UniProt names (for full species names see Figure 1D legend). **F.** *Left:* Structural alignment of Raf2 RFTS domain (pink) with murine DNMT1 (grey). *Right:* Structural alignment of Raf2 RFTS domain with human DNMT1 (grey).

Previous studies examined the localisation of GFP-Raf2 over-expressed from the strong *nmt1* promoter and demonstrated that Raf2 localises to both the nucleus and cytoplasm [43]. The observed cytoplasmic localisation could indicate that Raf2 has additional functions apart from its role within the CLRC complex function in the nucleus. However, the localisation of over-expressed Raf2 is not a robust test of its true subcellular location. In order to rigorously examine Raf2 localisation we utilized cells expressing Raf2 fused at its N-terminus to GFP (GFP-Raf2) from its own endogenous promoter and locus. We found that GFP-Raf2 localises predominantly to the nucleus in several distinct foci a subset of which colocalise with the centromere specific histone CENP-A^Cnp1^ at centromeres in the majority of cells examined (Figure 1A). This centromeric localisation of GFP-Raf2 is in agreement with previous genome-wide ChIP-on-Chip studies which indicate that like other CLRC components, Raf2 associates primarily with domains of heterochromatin [18]. We conclude that Raf2 is a heterochromatin-associated protein that mainly functions along with other CLRC subunits at centromeres.

During interphase the three fission yeast centromeres cluster adjacent to the SPB at the nuclear periphery. Cells lacking Raf2 (*raf2Δ*) have been reported to mislocalise GFP-tagged CENP-A^Cnp1^ to sites other than the centromere in approximately 20% of cells. Consequently Raf2 has been implicated in promoting CENP-A^Cnp1^ localisation to centromeres [26]. However, in wild type and *raf2Δ* cells we find that the localisation of untagged CENP-A^Cnp1^, detected with specific anti-Cnp1 antisera, is unchanged; a single focus of CENP-A^Cnp1^ fluorescent signal is detected in 100% of wild-type and *raf2Δ* cells. This suggests that CENP-A^Cnp1^ association with centromeres is not affected by loss of Raf2 (Figure 1B). Moreover, anti-Cnp1 chromatin immunoprecipitation (ChIP) shows that the levels of CENP-A^Cnp1^ associated with the central kinetochore domain at fission yeast centromeres in *raf2Δ* and wild-type cells are comparable (Figure 1C). If Raf2 has a specific role in directing CENP-A^Cnp1^ to centromeres, in addition to its role in heterochromatin integrity, cells lacking Raf2 may be expected to exhibit a greater level of chromosome segregation defect and cell inviability compared with *clr4Δ* cells, due to defective kinetochore function. However, several studies indicate that although Raf2/CLRC ensures accurate chromosome segregation by mediating heterochromatin formation and thus tight sister-centromere cohesion, cells lacking Raf2 do not exhibit a dramatic reduction in cell viability as might be expected if kinetochore integrity was disrupted [19], [21], [43]. Moreover, it is clear that cells lacking Raf2 do not display a more severe chromosome segregation defect than mutants, such as *clr4Δ*, that completely lack heterochromatin but do not affect the maintenance of Cnp1^CENP-A^ at pre-existing centromeres [44], [45].

### Raf2 shares similarity to DNMT1 through an RFTS domain

The *raf2* gene encodes a 73.29 kD protein containing a conserved N-terminal Replication Focus Targeting Sequence (RFTS) domain and an atypical C2H2 type zinc finger motif (Figure 1D). Raf2 has several canonical fungal orthologs (Figure S1A), and its RFTS domain shares 23% sequence identity with the RFTS domain of the maintenance DNA methyltransferase DNMT1. This similarity is underscored by the fact that the Raf2 RFTS domain can be structurally modelled on the RFTS domain from DNMT1 (Figure 1E and F). The architecture of other proteins containing RFTS domain are mainly of two types: those associated, and those not associated with a methyltransferase domain. RFTS domain proteins lacking methyltransferase domain are specific to fungi (Figure S1A) and the four species of fission yeast (*S. pombe*, *S. octosporus*, *S.cryophilus* and *S. japonicus*) only encode RFTS proteins devoid of a methyltransferase domain. The genomes of other fungi such as *Coccoides immitis* encode both RFTS only and RFTS plus methyltransferase domain proteins (Figure S1A). The majority of these fungal proteins have been identified though homology and remain uncharacterised. Indeed, despite the fact that Raf2 is required for heterochromatin integrity in *S. pombe* the role of the RFTS and its contribution to heterochromatin integrity and centromere function has not been investigated.

### Marker gene silencing is disrupted by mutations within the RFTS domain of Raf2

To determine the role of the Raf2 RFTS domain, we first deleted the entire domain (residue M1 to N203) from C-terminally FLAG-tagged Raf2 expressed from its own locus. However, the resulting Raf2-RFTSΔ-FLAG protein was unstable and the expected 52.6 kD band was not detectable by western (Figure S1B). Therefore, to study the function of RFTS, we introduced specific point mutations into residues conserved between both fungal (I98A) or mammalian proteins (E104A).

In addition to mutating these residues, we previously isolated a conditional temperature sensitive (ts) allele, *raf2-1*, in a screen for mutants which disrupt heterochromatin at 36°C, but not at 25°C [23]. The *raf2-1* mutant expresses a protein with a missense mutation (S100F) in a conserved fungal residue within the RFTS domain, known hereafter as *raf2-S100F*. These three mutated residues, I98, S100 and E104, reside in a flexible looped region between the main α-helix of the RFTS domain and the β-sheet region (Figure S1C).

**Figure 2:**
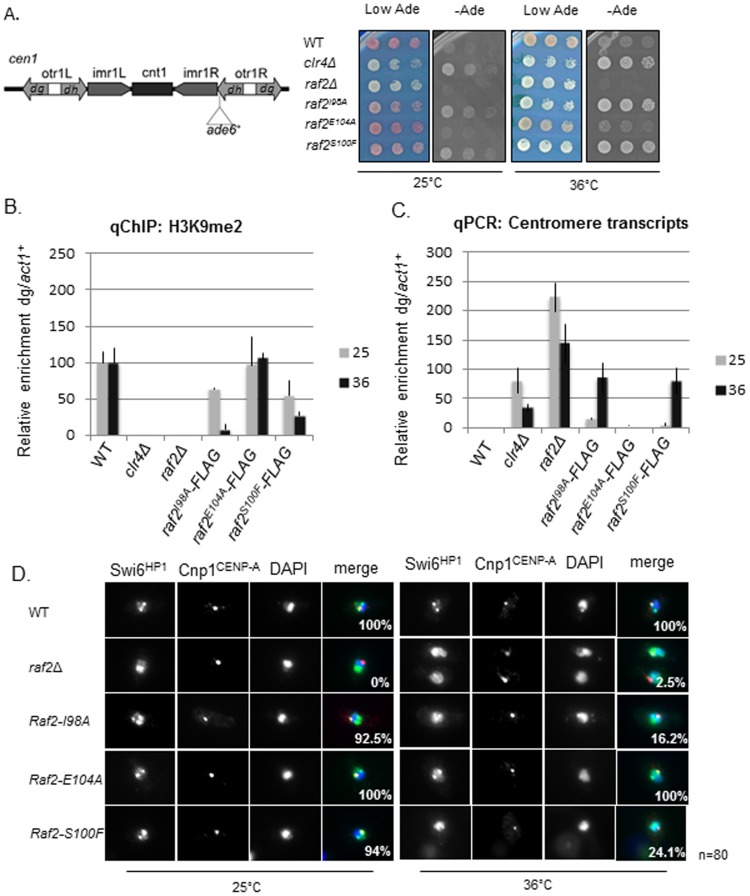
RFTS mutants conditionally disrupt heterochromatin integrity. **A.** Assay for silencing at *cen1:ade6+*. Diagram shows the position of the *cen1:ade6+* marker gene within centromere 1, relative to the outer repeat (otr) *dg* and *dh* elements, innermost repeats (*imr*), and central core (*cnt*). Wild-type cells with the marker gene repressed form red colonies on low adenine whereas cells with disrupted heterochromatin such as *clr4Δ* or *raf2Δ* cause marker gene expression and form white colonies. *Raf2-I98A* and *raf2-S100F* disrupt marker gene silencing specifically at 36°C but not at 25°C. **B.** ChIP analysis of H3K9me2 levels associated with *cen(dg)* relative to *act1+* in *clr4Δ* and raf2 mutant cells normalised to wild-type at 25°C or 36°C. Error bars: SEM. **C.** qRT-PCR analysis of *cen(dg)* transcript levels relative to a control transcript *act1+*, normalised to wild-type at 25°C or 36°C. Error bars: SEM. **D.** Swi6 localisation in wild-type or mutant cells at 25°C or 36°C. Representative images of fixed cells with Swi6 (green), CENP-A^Cnp1^(red) and DNA (DAPI-blue). Numbers shown denote % cells with Swi6 localised at centromeres, as determined by colocalisation with Cnp1, from a total number of 80 cells per sample.

Marker genes inserted at silent heterochromatic loci are transcriptionally repressed and are sensitive to defects in heterochromatin components [8], [46]. We tested marker gene silencing within centromeric outer repeats (*cen1:ade6^+^*) (Figure 2A). Both the *raf2-I98A* and *raf2-S100F* mutations alleviate silencing of the *ade6^+^* gene inserted at this location at 36°C, but not 25°C, whereas *raf2-E104A* does not (Figure 2A). Importantly, western analysis indicates that none of these point mutations affect the levels of Raf2 protein produced (Figure S2A). These analyses indicate that conserved residues of the RFTS domain are important for Raf2 function in forming robust heterochromatin.

### Heterochromatin integrity is compromised by mutations within the RFTS domain of Raf2

Cells lacking any of the CLRC components (*cul4Δ clr4Δ*, *rik1Δ*, *raf1Δ* or *raf2Δ*) display loss of H3K9 methylation and delocalisation of Swi6 [18], [19], [21], [23], [43]. ChIP analysis indicates that the level of H3K9 methylation on centromeric repeats in all three RFTS domain mutants was similar to wild-type cells at 25°C. However, in keeping with perturbed silencing of marker genes, H3K9 methylation levels were significantly reduced at 36°C in *raf2-I98A* and *raf2-S100F* cells (Figure 2B). Importantly, Clr4 protein levels were unaffected by mutations in the RFTS domain of Raf2 thus the reduction of H3K9 methylation was not due to a loss of Clr4 (Figure S2B).

Deletion of components involved in centromeric heterochromatin formation exhibit accumulation of non-coding centromere repeat transcripts as transcription is no longer repressed [12], [47]. In accordance with this, centromere transcript levels were observed to be comparable with *clr4Δ* cells at 36°C in *raf2-I98A* and *raf2-S100F* cells, whereas centromere transcripts in cells bearing the *raf2-E104A* allele were similar to wild-type (Figure 2C).

A reduction in H3K9 methylation is expected to perturb Swi6 localisation. The proportion of cells with Swi6 localized to centromeres was found to be greatly reduced in *raf2-I98A* and *raf2-S100F* mutants at 36°C compared to wild-type cells (Figure 2D). As expected, the *raf2-E104A* allele did not affect Swi6 localisation.

**Figure 3:**
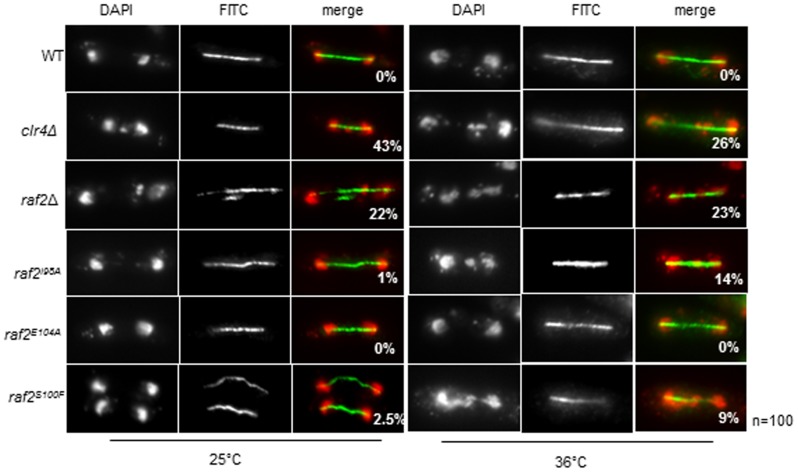
Raf2 conditional mutants display defective chromosome segregation at the restrictive temperature. Analysis of lagging chromosomes in anaphase by fluorescence microscopy. Cells with defective heterochromatin display lagging chromosomes in anaphase. Shown here are representative images of fixed cells stained with DAPI (red) and tubulin (green). The numbers shown denote % anaphase cells with lagging chromosomes. 100 mitotic cells were counted for each sample.

Disruption of centromeric heterochromatin is known to result in defective chromosome segregation as seen in *clr4Δ* and RNAi mutants [44], [47]. In concordance with the observed defects in centromeric heterochromatin integrity, a high frequency of lagging chromosomes was evident in late anaphase *raf2-I98A* and *raf2-S100F* cells at 36°C, but not in *raf2-E104A* or wild-type cells (Figure 3).

In summary, the above analyses indicate that the RFTS domain of Raf2 is important for the maintenance of heterochromatin integrity and thus centromere function.

### siRNA production is maintained in RFTS mutants

**Figure 4:**
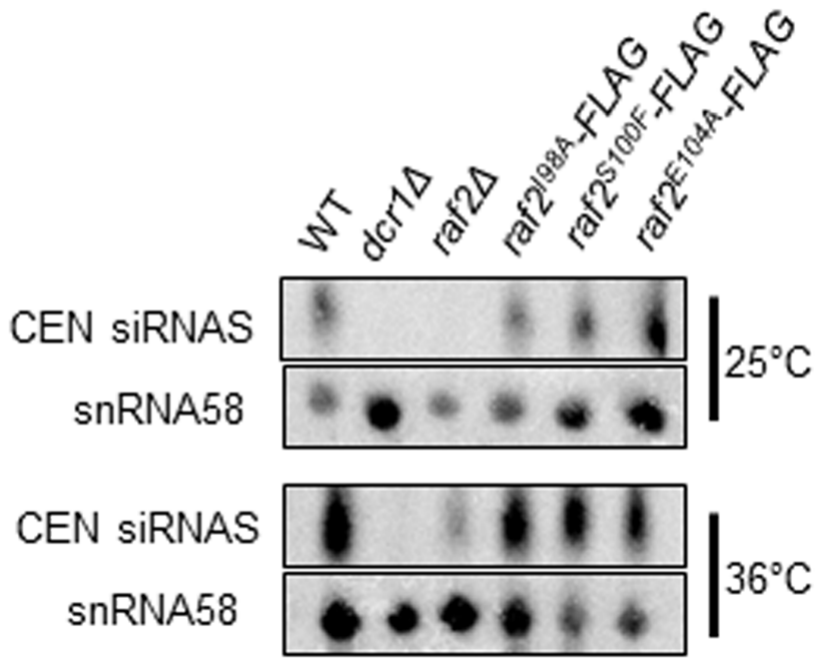
Raf2 RFTS mutants can generate siRNAs. Northern blot analysis of centromeric siRNAs at 25°C and 36°C. snoRNA58 (snR58) is shown as a loading control. In wild-type cells, siRNAs are generated from centromeric repeats, but RNAi mutants lack the ability to process precursor RNAs.

The CLRC complex is required for siRNA production [13], [21]. However, it has been shown that specific point mutations in the CLRC components Raf1 and Cul4 cause disruption of heterochromatin whilst siRNA generation is unaffected [23], [48]. To determine whether siRNA generation is disrupted by mutations within the RFTS domain northern analysis was performed. We observed that, unlike *dcr1*Δ and *raf2*Δ mutants, centromeric siRNAs are produced at wild-type levels in *raf2-I98A*, *raf2-S100F and raf2-E104* cells at both 25°C and 36°C (Figure 4). Therefore, it appears that, as reported for specific *raf1* and *cul4* mutants, point mutants within the RFTS domain of Raf2 uncouple siRNA production from H3K9 methylation. These analyses provide additional support for the finding that CLRC is absolutely required for H3K9 methylation at centromeres but is dispensable for the production of siRNA, as previously proposed [23], [48].

### Mutations within the RFTS domain disrupt the interaction of Raf2 with Cul4

The function of the RFTS domain of mammalian DNMT1 has been the subject of several studies [27], [32], [39], [49]. However, the role of RFTS domains in proteins such as Raf2 in organisms like *S. pombe* that lack DNA methylation remains unknown.

**Figure 5:**
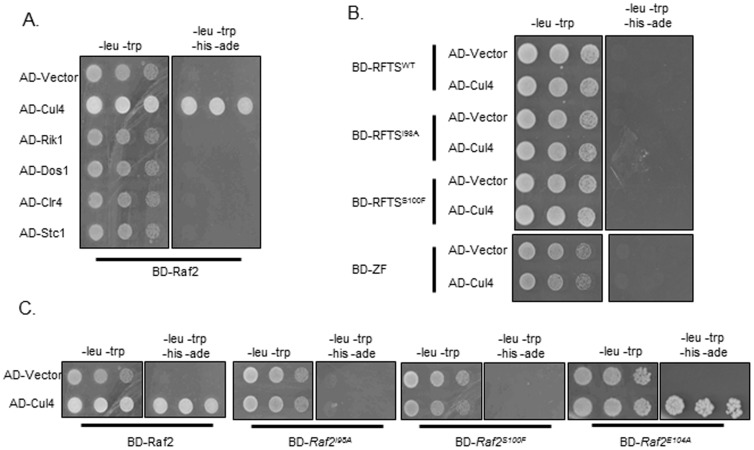
Raf2 mutations disrupt interactions with Cul4 but not Cdc20. **A.** Yeast -2-hybrid assay. Interaction of Raf2 with Cul4 is indicated by growth on -Leu, -Trp, -His, -Ade plates. BD and AD: GAL4 *B*inding or *A*ctivation *D*omain fusions, respectively. **B.** Both the RFTS domain and zinc finger domain are required for interaction with Cul4. **C.** Specific point mutations within the RFTS domain disrupt the interaction of Raf2 with Cul4.

As point mutations within the Raf2 RFTS domain affect heterochromatin integrity we tested whether these specific *raf2* mutations affect the interactions between Raf2 and other components of CLRC. We therefore set up a targeted yeast-two-hybrid (Y2H) assay to determine if the *Raf2-I98A*, *Raf2-S100F* and *Raf2-E104* mutations affect interactions between Raf2 and other CLRC components. As previously described, we detect a direct interaction between full-length Raf2 and Cul4 (Figure 5A) [22] while interactions of Raf2 with Rik1, Raf1 and Clr4 could not be detected by Y2H. Our Y2H analyses demonstrate that the *Raf2-I98A* and *Raf2-S100F* mutations, but not the weak *Raf2-E104* mutation, disrupt the Raf2-Cul4 interaction (Figure 5C). Further Y2H assays indicate that neither the RFTS domain nor the zinc finger domain of Raf2 alone were sufficient to mediate the interaction with Cul4 (Figure 5B). These data demonstrate that the RFTS domain is necessary, but not sufficient, for integrating Raf2 within the CLRC complex suggesting that the overall tertiary structure of full-length Raf2 may be important to mediate the Raf2-Cul4 interaction (Figure 5B).

## Discussion

### Raf2 is a heterochromatin protein that is not involved in maintaining CENP-A at centromeres

In this study, we have analysed the function and localisation of the CLRC component Raf2. Expression of GFP-Raf2 tagged protein expressed at endogenous levels, demonstrates that Raf2 is a truly heterochromatic protein with predominantly nuclear localisation as previously documented [43]. Raf2 has been recently reported to be involved in the maintenance of the key centromere specific histone CENP-A at centromere [26]. However, that study utilised strains expressing C-terminally GFP-tagged CENP-A^Cnp1^ which is not fully functional [50]. Moreover, it is well established that Raf2 is required for heterochromatin integrity but is not essential for cell viability, CENP-A mislocalisation would be expected to have a greater impact on cell viability [19], [21], [48]. Here we have utilised antisera specific for Cnp1^CENP-A^ for immunolocalisation and show that endogenous untagged Cnp1^CENP-A^ localisation remains at centromeres in all cells lacking Raf2. We also do not detect a drop in the level of Cnp1^CENP-A^ associated with central CENP-A chromatin domain of centromeres in *raf2Δ* cells in ChIP analyses. Overall our analyses demonstrate that Raf2 is predominantly a nuclear protein that functions within the CLRC complex to mediate heterochromatin formation and is not required to maintain CENP-A with the central domain of centromeres.

### The Raf2 RFTS domain is required for heterochromatin integrity

Raf2 is a subunit of the Cul4 dependent CLRC E3 ubiquitin ligase, whose *in vitro* substrates remain unknown [23], [26]. We have demonstrated that that Raf2 RFTS domain can be structurally aligned with the RFTS domain of the human DNA methyltransferase DNMT1. In addition, we show that the Raf2 RFTS is required for centromeric heterochromatin integrity. Fission yeast cells carrying specific point mutations in the RFTS domain are defective in centromeric heterochromatin formation and function.

It is important to note that, like Raf2, DNMT1 has been shown to localise to pericentric heterochromatin in mammalian cells. In addition, its stability is regulated by the action of an E3 ubiquitin ligase, UHRF1, and a de-ubiquitinase, Usp7 [33]. We suggest that the RFTS domain is a protein module that may be used in many distinct systems to couple chromatin/DNA modifiers to ubiquitin ligase activity. In support of this hypothesis, we find that mutations within the RFTS domain impair the interaction between Raf2 and the cullin Cul4, the essential scaffold component for a bona-fide Cullin-dependent E3 ubiquitin ligase. This Raf2 RFTS-Cul4 interaction may be essential for the integrity of CLRC and its ability to direct or regulate the methylation of histone H3K9 by the Clr4 methyltransferase.

### The Raf2 RFTS domain is required for heterochromatin integrity but not siRNA generation

**Figure 6:**
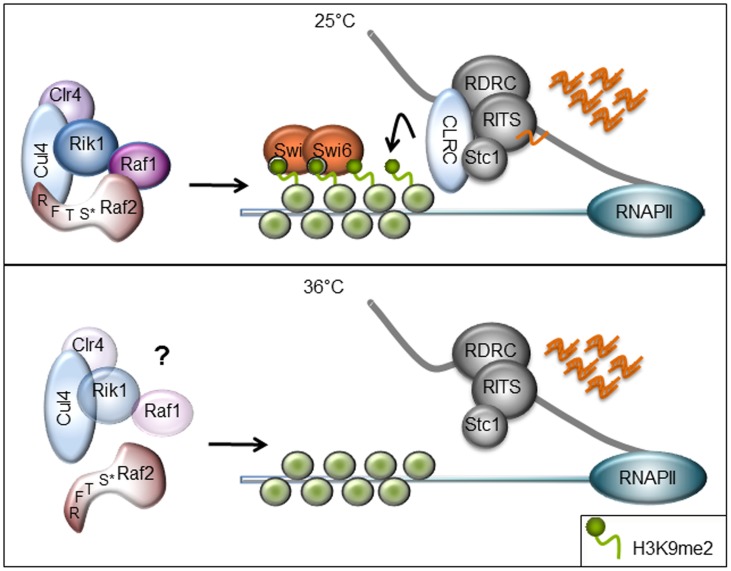
Schematic diagram of heterochromatin defect in Raf2 RFTS mutants. Cells containing point mutations within the RFTS domain of Raf2 maintain an intact CLRC at 25°C, siRNAs are generated from non-coding RNA transcripts originating from the centromere and chromatin modifications are targeted back to homologous regions. At 36°C, the point mutations cause a conformational change within Raf2 and interfere with its interaction with Cul4. In disrupting CLRC interactions, the Raf2 RFTS mutants cause loss of H3K9 methylation as Clr4 may no longer be targeted to chromatin.

CLRC has two major functions in heterochromatin formation: it possesses histone methyltransferase activity via Clr4 and mediates siRNA production [21], [51]. In wild-type fission yeast, these processes are coupled to direct heterochromatin formation to specific location such as centromeres, telomeres and the silent mating-type locus, and prohibit silencing elsewhere (Figure 6). Cells expressing only mutant histone H3 (H3K9R) are unable to methylate K9 of H3 and do not form heterochromatin, however such cells continue to produce a low level of siRNAs homologous to centromeric repeats [52], [53]. This suggests that the CLRC complex plays a role in promoting siRNA production, independently of H3K9 methylation.

Deletion of any CLRC component results in loss of both H3K9 methylation and siRNA production, yet point mutations within CLRC components Raf1 and Cul4 exhibit separable functions with respect to chromatin modification and siRNA generation [23], [48]. We demonstrate here that specific mutations within the RFTS domain of Raf2 result in the loss of the classic marks of heterochromatin, namely H3K9 methylation and Swi6, but maintain siRNA production. Thus, as previously documented for specific mutations within Raf1 and Cul4, mutation in the RFTS domain of Raf2 uncouple chromatin modification from siRNA production [23], [48]. This effect may be due to partial disruption of the CLRC; the point mutants studied may be able to maintain specific interactions required for siRNA generation but lose those that are critical for H3K9 methylation and subsequent protein associations. It may be that, as seen in specific Raf1 mutants, siRNA levels remain high because the defective Raf2 RFTS mutations inhibit the degradation of pre-existing siRNAs [23]. Another tenable explanation is that the particular Raf2 RFTS mutants analysed do not disrupt the continual synthesis of siRNAs from centromere repeat transcripts. In fact, since Raf2 has been shown to interact with Cdc20, this could provide a molecular link between DNA replication, siRNA production and chromatin modification [25]. More extensive analyses of such interactions in cells harboring mutations such as those in the Raf2 RFTS mutation should provide insight into the interplay between Raf2, Cdc20 and the role of DNA replication in these processes and allow further dissection of the role of the CLRC complex in RNAi-directed heterochromatin formation.

## Supporting Information

Figure S1 **A.** Multiple sequence alignment of Raf2 fungal homologous proteins. The amino acid coloring scheme indicates average BLOSUM62 scores (which are correlated with amino acid conservation) for each alignment column: red (greater than 3), violet (between 3 and 1.5) and light yellow (between 1.5 and 0.5). Sequences are named according to their UniProt names. Full species names are: RAF2_SCHPO, *Schizosaccharomyces pombe*; S9X856_SCHCR, *Schizosaccharomyces cryophilus*; B6K1K8_SCHJY, *Schizosaccharomyces japonicus*; L8FMY4_PSED2, *Pseudogymnoascus destructans*; G2Q7S7_THIHA, *Thielavia heterothallica*; B2B7F9_PODAN, *Podospora anserina*; G4ML14_MAGO7, *Magnaporthe oryzae*; C1GEE7_PARBD, *Paracoccidioides brasiliensis*; J3K2M4_COCIM, *Coccidioides immitis*; A2Q7V8_ASPNC, *Aspergillus niger*; D4DHS5_TRIVH, *Trichophyton verrucosum*; K2RLQ8_MACPH, *Macrophomina phaseolina*. Residues which are subject to mutation are labeled. RFTS and C2H2 Zinc Finger domains are boxed in violet and red, respectively. **B.** Raf2 protein missing the entire RFTS domain does not encode a truncated protein. **C.** Zoom-in of RFTS structure showing the region containing the point mutations.
(TIF)

Figure S2 **A.** Western blot demonstrating that both wild type and proteins containing point mutations within the RFTS domain are produced at 36°C. **B.** Clr4 levels remain constant in cells containing point mutations within the RFTS domain of Raf2. TAT1 is shown as a loading control.
(TIF)

Figure S3 Western blots demonstrating expression of yeast-2-hybrid proteins.
(TIF)

Table S1 List of *S.pombe* strains used in this study.
(DOCX)

Table S2 List of primers used in this study.
(DOCX)
