# Supplementary figures and images for: The RFTS Domain of Raf2 Is Required for Cul4 Interaction and Heterochromatin Integrity in Fission Yeast
Source: PLoS One. 2014 Aug 4;9(8):e104161. doi: 10.1371/journal.pone.0104161 (PMC4121317; doi:10.1371/journal.pone.0104161)

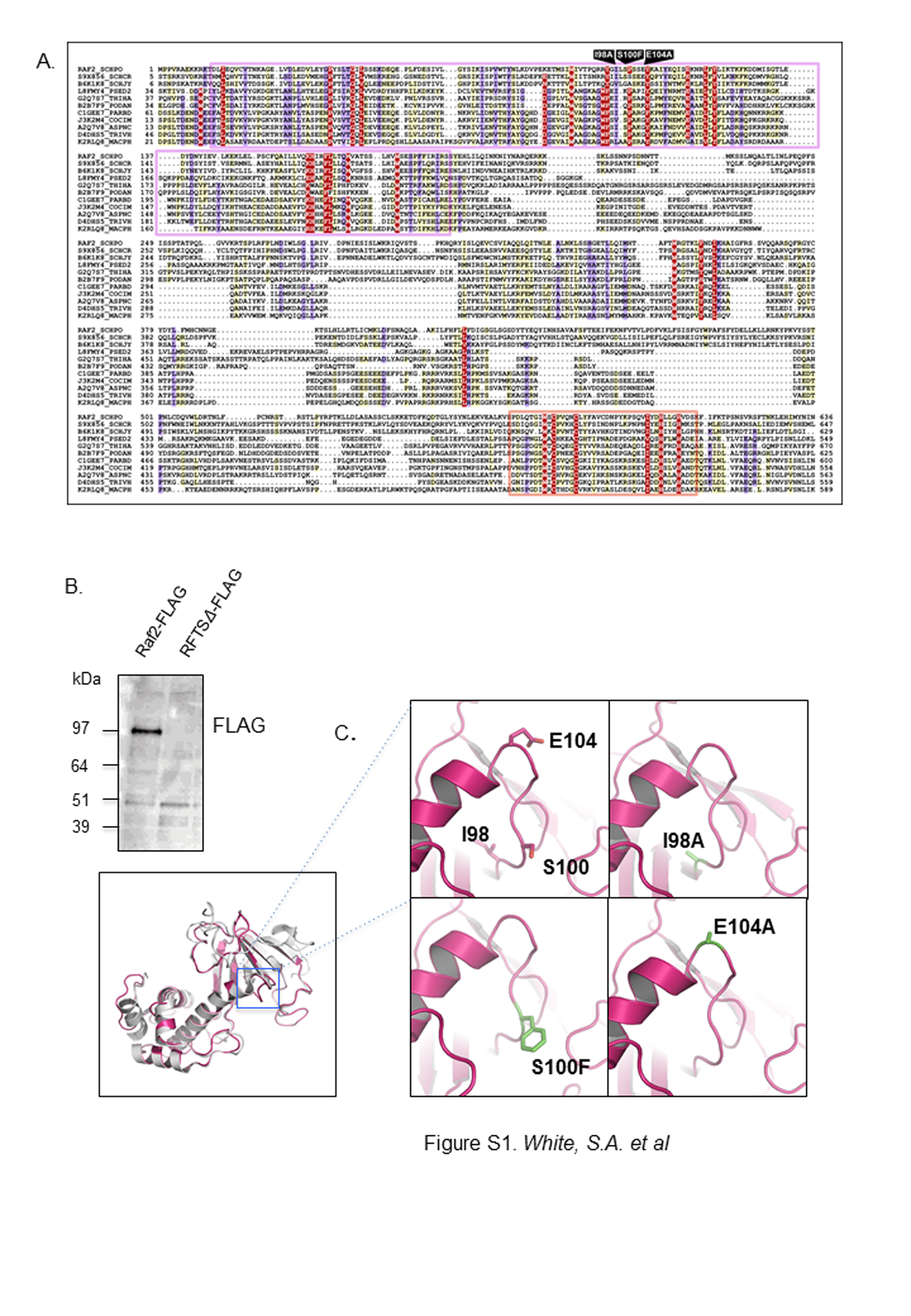

Supplement: Figure S1 — A. Multiple sequence alignment of Raf2 fungal homologous proteins. The amino acid coloring scheme indicates average BLOSUM62 scores (which are correlated with amino acid conservation) for each alignment column: red (greater than 3), violet (between 3 and 1.5) and light yellow (between 1.5 and 0.5). Sequences are named according to their UniProt names. Full species names are: RAF2_SCHPO, Schizosaccharomyces pombe; S9X856_SCHCR, Schizosaccharomyces cryophilus; B6K1K8_SCHJY, Schizosaccharomyces japonicus; L8FMY4_PSED2, Pseudogymnoascus destructans; G2Q7S7_THIHA, Thielavia heterothallica; B2B7F9_PODAN, Podospora anserina; G4ML14_MAGO7, Magnaporthe oryzae; C1GEE7_PARBD, Paracoccidioides brasiliensis; J3K2M4_COCIM, Coccidioides immitis; A2Q7V8_ASPNC, Aspergillus niger; D4DHS5_TRIVH, Trichophyton verrucosum; K2RLQ8_MACPH, Macrophomina phaseolina. Residues which are subject to mutation are labeled. RFTS and C2H2 Zinc Finger domains are boxed in violet and red, respectively. B. Raf2 protein missing the entire RFTS domain does not encode a truncated protein. C. Zoom-in of RFTS structure showing the region containing the point mutations. (TIF) [file pone.0104161.s001.tif]

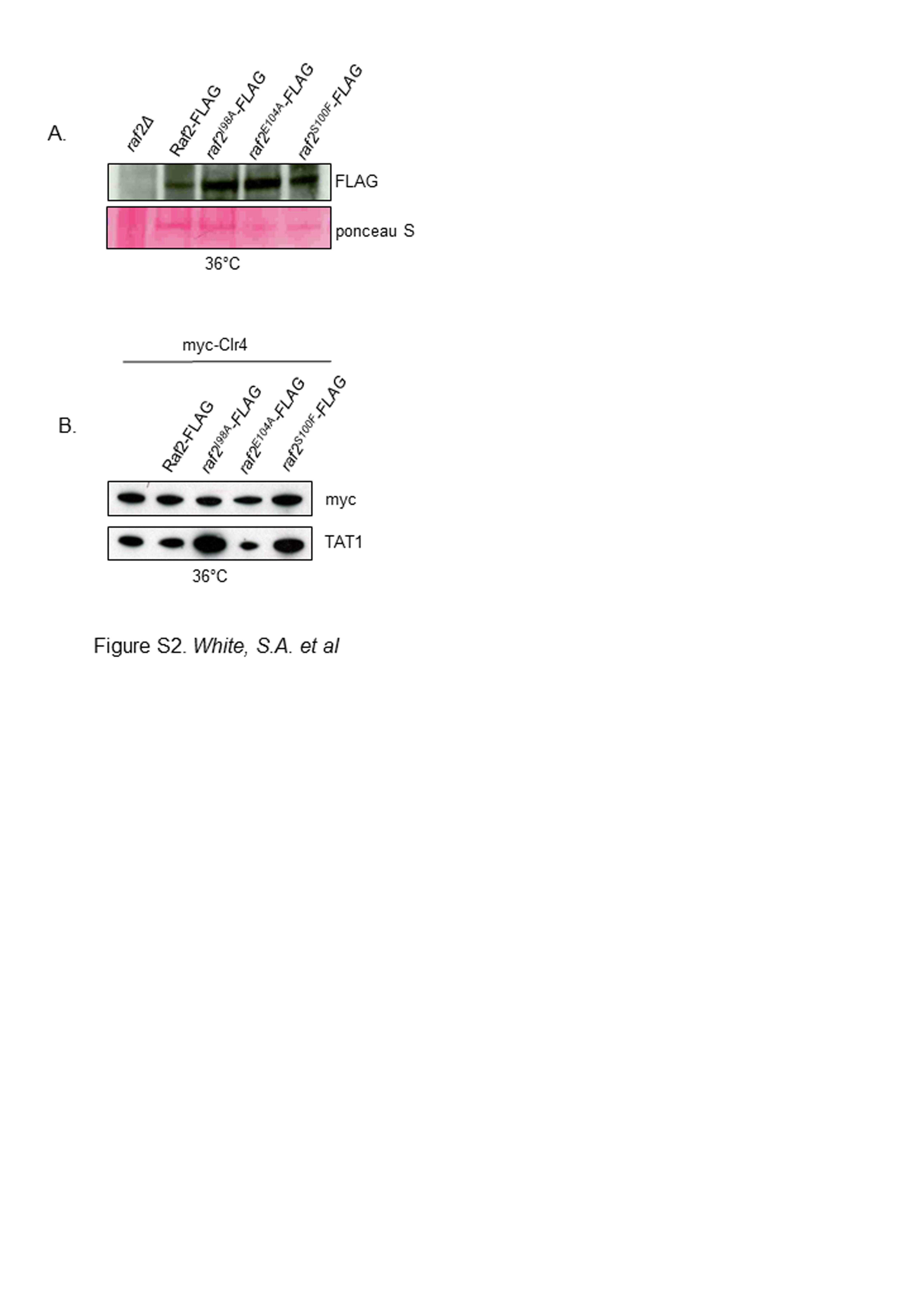

Supplement: Figure S2 — A. Western blot demonstrating that both wild type and proteins containing point mutations within the RFTS domain are produced at 36°C. B. Clr4 levels remain constant in cells containing point mutations within the RFTS domain of Raf2. TAT1 is shown as a loading control. (TIF) [file pone.0104161.s002.tif]

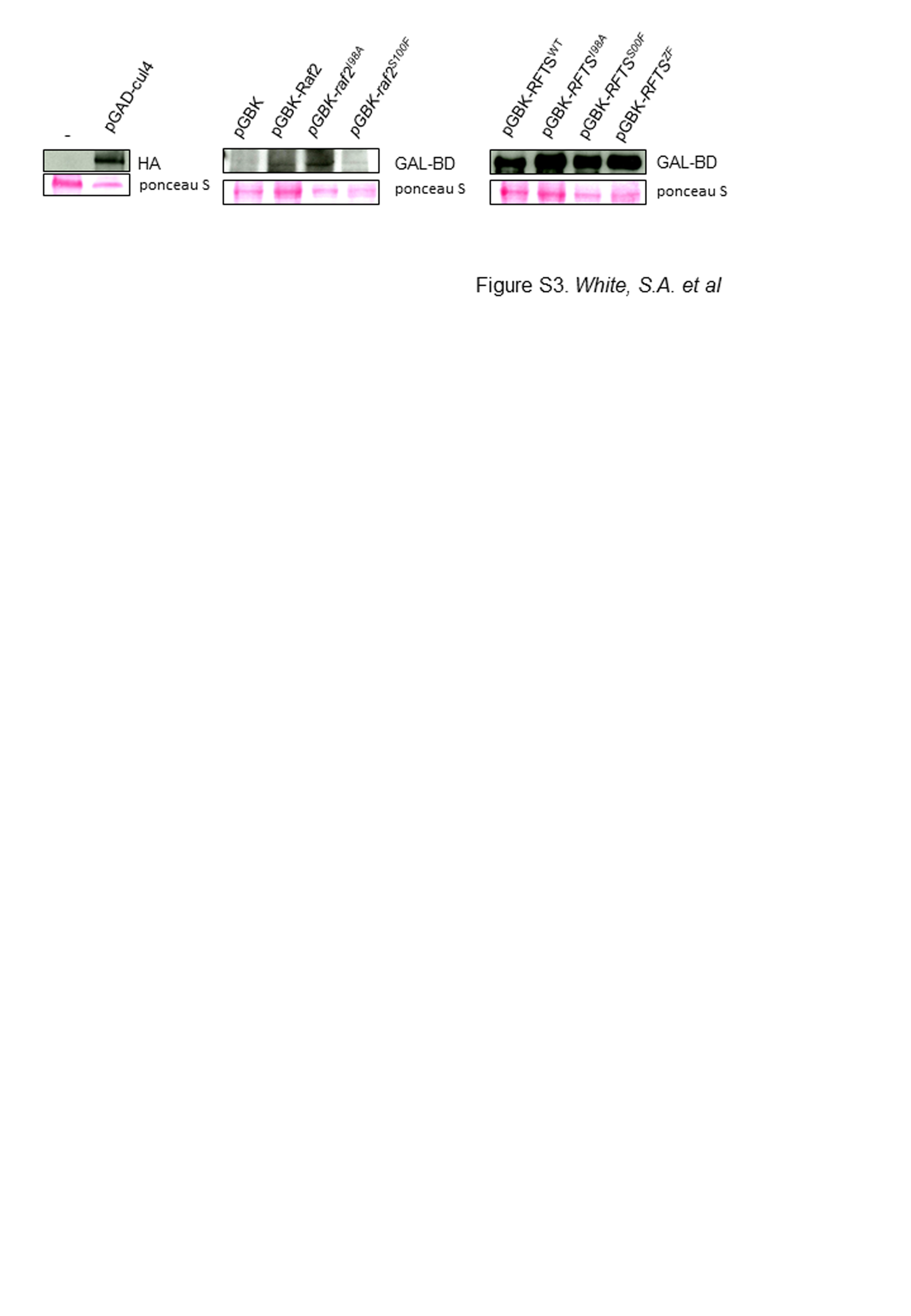

Supplement: Figure S3 — Western blots demonstrating expression of yeast-2-hybrid proteins. (TIF) [file pone.0104161.s003.tif]
