# Supplementary material for: The RFTS Domain of Raf2 Is Required for Cul4 Interaction and Heterochromatin Integrity in Fission Yeast
Source: PLoS One. 2014 Aug 4;9(8):e104161. doi: 10.1371/journal.pone.0104161 (PMC4121317; doi:10.1371/journal.pone.0104161)
Supplement: Table S1 — List of S.pombe strains used in this study. (DOCX) [file pone.0104161.s004.docx]

| ***Table S1.*** *List of strains used in this study* | |
| --- | --- |
| FY 1180 | h+ ade6-210 leu1-32 ura4-D18 otr1R (dg-glu) Sph1::ade6 |
| FY 8138 | h- raf2::KAN leu1-32 ade6-210 ura4-D18 his3D1 |
| FY8635 | h+ Nmyc-clr4 ade6-210 leu1-32 ura4D18 otr1RSph::ade6 |
| FY 8815 | h- raf2::KanMX cen1otr(Sph1):ade6+ lys1:Natr leu1-32 ade6-210? ura4D18 |
| FY 8898 | hA clr4:KanMX ade6-210 leu1-32 ura4 lys1:Nat cen1otr1(Sph):ade6 |
| FY 14191 | h+ raf2-FLAG ade6otr ade6-210 leu1-32 ura4-D18 |
| FY 14360 | h- GFP-Raf2::ura4+ leu1-32 ura4-D18 |
| FY 15059 | h- raf2-ts3-Flag-NatR ade6-210 leu1-32 ura4-D18 otr Sph:ade6 |
| FY 17087 | h+ raf2-RFTS::ura4-FLAG otr1RSph1-ade6 ade6-210 leu1-32 ura4D18 |
| FY 18160 | h+ raf2-I98A-FLAG leu1-32 ura4D18 ade6-210 ade6(SphI)otr1 |
| FY 18161 | h+ raf2-E104A-FLAG leu1-32 ura4D18 ade6-210 ade6(SphI)otr1 |
| FY18796 | h? raf2-I98A-FLAG-NAT Nmyc-clr4 leu1-32 |
| FY18797 | h? raf2-I98A-FLAG-NAT Nmyc-clr4 leu1-32 |
| FY18798 | h? raf2-S100F-FLAG-NAT Nmyc-clr4 leu1-32 |
| FY18799 | h? raf2-S100F-FLAG-NAT Nmyc-clr4 leu1-32 |
