# Supplementary material for: The RFTS Domain of Raf2 Is Required for Cul4 Interaction and Heterochromatin Integrity in Fission Yeast
Source: PLoS One. 2014 Aug 4;9(8):e104161. doi: 10.1371/journal.pone.0104161 (PMC4121317; doi:10.1371/journal.pone.0104161)
Supplement: Table S2 — List of primers used in this study. (DOCX) [file pone.0104161.s005.docx]

| ***Table S2.*** *List of primers used in this study* | |
| --- | --- |
| **Primer name** | **Sequence 5'-3'** |
| **Northern probes** |  |
| IK8 | ATTCCTTTCTGAACCTCTCTGTTAT |
| IK9 | TTTGATGCCCATGTTCATTCCACTTG |
| IK10 | GGGAGTACATCATTCCTACTTCGATA |
| snR58 | GATGAAATTCAGAAGTCTAGCATC |
| **qPCR** |  |
| q_cen(dg)_F | AATTGTGGTGGTGTGGTAATAC |
| q_cen(dg)_R | GGGTTCATCGTTTCCATTCAG |
| q_act_F | GGTTTCGCTGGAGATGATG |
| q_act_R | ATACCACGCTTGCTTTGAG |
| q-cnt1-F | CAGACAATCGCATGGTACTATC |
| q-cnt1-R | AGGTGAAGCGTAAGTGAGTG |
| **Mutagenesis and deletion** |  |
| RFTSURAF | TCGAAGAGTATTGTTTTTGGGGAAAAGGAAAAAGCTAGCGATGCCGCCCGTACGTGCTGAAAAAAGCGGAAAACTGATTATCCCACTGGCTATATGTATGC |
| RFTSURAR | ATAGTAGTATTATTGTCCGATGGATTGTTTGACGATAACTTTGATTTCTTTCGCTCTTGACGAGCATTGTAAATGTTTTTCATCCAACACCAATGTTTATAACCAAG |
| E104F | AATTTTGTCTCCTTCTTCTGCGTATAAAGCCATTTACGAA |
| E104R | TTCGTAAATGGCTTTATACGCAGAAGAAGGAGACAAAATT |
| I98AF | ACACCTCAAAGAAGATACGGAGCTTTGTCTCCTTCTTCTG |
| I98R | CAGAAGAAGGAGACAAAGCTCCGTATCTTCTTTGAGGTGT |
| **Yeast-2-hybrid** |  |
| BamHI-Raf2F | GGATCCATGCCGCCCGTACGTGCTGA |
| SalI-Raf2R | GTCGACCCTGTTGTACATTATGTGTTCA |
| Sal-RFTSR | GCGTCGACATTAATTTGTAAAATCAGAT |
| BamHI-ZF | GCGGATCCAAAAACATTTACAATGCTCG |
